# Use of genomic information to exploit genotype-by-environment interactions for body weight of broiler chicken in bio-secure and production environments

**DOI:** 10.1186/s12711-019-0493-3

**Published:** 2019-09-18

**Authors:** Thinh T. Chu, John W. M. Bastiaansen, Peer Berg, Hélène Romé, Danye Marois, John Henshall, Just Jensen

**Affiliations:** 1 Center for Quantitative Genetics and Genomics, Department of Molecular Biology and Genetics, Aarhus University, 8830 Tjele, Denmark; 2 Wageningen University & Research, Animal Breeding and Genomics, 6709 PG Wageningen, The Netherlands; 3 Faculty of Animal Science, Vietnam National University of Agriculture, Gia Lam, Hanoi, Vietnam; 4 Department of Animal and Aquacultural Sciences, Norwegian University of Life Sciences, 1432 Ås, Norway; 5 Cobb-Vantress Inc, Siloam Springs, AR 72761-1030 USA

## Abstract

**Background:**

The increase in accuracy of prediction by using genomic information has been well-documented. However, benefits of the use of genomic information and methodology for genetic evaluations are missing when genotype-by-environment interactions (G × E) exist between bio-secure breeding (B) environments and commercial production (C) environments. In this study, we explored (1) G × E interactions for broiler body weight (BW) at weeks 5 and 6, and (2) the benefits of using genomic information for prediction of BW traits when selection candidates were raised and tested in a B environment and close relatives were tested in a C environment.

**Methods:**

A pedigree-based best linear unbiased prediction (BLUP) multivariate model was used to estimate variance components and predict breeding values (EBV) of BW traits at weeks 5 and 6 measured in B and C environments. A single-step genomic BLUP (ssGBLUP) model that combined pedigree and genomic information was used to predict EBV. Cross-validations were based on correlation, mean difference and regression slope statistics for EBV that were estimated from full and reduced datasets. These statistics are indicators of population accuracy, bias and dispersion of prediction for EBV of traits measured in B and C environments. Validation animals were genotyped and non-genotyped birds in the B environment only.

**Results:**

Several indications of G × E interactions due to environmental differences were found for BW traits including significant re-ranking, heterogeneous variances and different heritabilities for BW measured in environments B and C. The genetic correlations between BW traits measured in environments B and C ranged from 0.48 to 0.54. The use of combined pedigree and genomic information increased population accuracy of EBV, and reduced bias of EBV prediction for genotyped birds compared to the use of pedigree information only. A slight increase in accuracy of EBV was also observed for non-genotyped birds, but the bias of EBV prediction increased for non-genotyped birds.

**Conclusions:**

The G × E interaction was strong for BW traits of broilers measured in environments B and C. The use of combined pedigree and genomic information increased population accuracy of EBV substantially for genotyped birds in the B environment compared to the use of pedigree information only.

## Background

The difference in production conditions between highly bio-secure breeding (B) and commercial production environments (C) can lead to genotype-by-environment interaction (G × E) in broiler chicken [1]. Indications of G × E may include heterogeneous variances, different heritabilities, and correlations lower than 1 between the same trait expressed under the B and C conditions [1]. To model G × E, the same trait expressed in the two environments can be defined as two correlated traits. Identification of the presence of G × E, especially a genetic correlation between traits measured in B and C, is important for optimizing breeding programs. For example, Chu et al. [2] showed that the genetic correlation between traits measured in B and C environments can change the optimal proportion of birds to be tested in the B versus the C environment. For body weight (BW) of broiler chicken, G × E interactions have been reported with genetic correlations ranging from 0.46 to 0.69 [1], from 0.74 to 0.76 [3] and from 0.75 to 0.76 [4] between traits measured in environments B and C.

The ultimate goal of a breeding program for broilers is to increase the genetic gain of the birds’ performance in the C environment only. To improve genetic gain, sib-testing of purebred birds in environments B and C is an option [1]. Due to bio-security restrictions, only the birds in the B environment are selection candidates, whereas the birds in the C environment provide information on the performance in C only. Because of limited reproductive capacity of broiler dams, a restricted number of birds can be moved to the C environment for phenotype testing, and thus accuracy of prediction for performance in C might be relatively low with pedigree-based best linear unbiased prediction (PBLUP) [2]. In this situation, genomic information can be of interest to improve accuracy of prediction.

There has been growing interest for genomic selection in poultry breeding programs because of the higher accuracy of prediction compared to pedigree-based evaluation. The increase in accuracy of prediction from using dense genotypes is due to improved measurement of the relationships between animals and better prediction of the Mendelian sampling terms [5]. Improved knowledge of the relationships may help the transfer of information from birds in the C environment to selection candidates in the B environment. The benefit of genomic selection over pedigree-based selection has been well documented from simulations [5–10] and empirical studies in chicken [11–15], cattle [16–19] and pig [10, 20–22] breeding schemes. However, none of the empirical studies reported a benefit of the use of genomic information in a breeding program with G × E sib-testing designs. In such a program, a multivariate joint model is required to model traits measured in commercial and breeding environments. When the number of genotyped individuals and single nucleotide polymorphisms (SNPs) are large, the use of a multi-trait model can be computationally challenging, and estimation of variance components from the model that uses a realized genomic relationship matrix can be tedious. Model-based accuracy or individual accuracy of estimated breeding values (EBV) cannot be computed because it is not feasible to obtain prediction error variances by direct inversion of the left hand side (LHS) of mixed model equations. Cross-validation strategies for accuracy of EBV based on correlations between EBV and corrected phenotypes cannot be applied. In a G × E sib-testing breeding program, validation animals are the birds in the B environment, which do not have corrected phenotypes for the traits measured in environment C. Legarra and Reverter [23] proposed cross-validation measures that can compare competing prediction models in situations of breeding programs where traits are influenced by G × E interactions. These validation measures [23] are based on statistics of EBV that are estimated from full and reduced datasets.

In addition, a typical breeding program that uses genomic selection in broiler chicken often applies a selective genotyping strategy. In broilers, important traits such as BW and feed efficiency can be recorded before sexual maturity. In situations of limited resources for genotyping, only a proportion of the birds that are potential parents with best performances will be genotyped. The selective genotyping strategy can increase accuracy of selection and genetic gain compared to random genotyping strategies [24]. However, the selective genotyping can create bias and lead to overestimation of genetic variances when single-step genomic BLUP (ssGBLUP) is used to estimate variance components [25]. To exploit genomic information, as well as pedigree data and phenotypes of non-genotyped birds in genetic evaluation, ssGBLUP models [22] can be used. However, accurate prediction of breeding values requires accurate estimates of variance components. An animal model using the pedigree relationship matrix is currently recommended to estimate variance components in the situation of selective genotyping [25].

Two main objectives of our study were: (1) to explore genotype-by-environment interactions for BW in broilers raised in breeding bio-secure (B) and commercial production (C) environments, and (2) to use genomic information to increase the accuracy of predicted breeding values of birds in the B environment for BW traits measured in the C environment.

## Methods

### Data

Data obtained from the poultry breeding company, Cobb-Vantress, included (BW) performances of purebred broiler chicken that were tested in breeding bio-secure (B) and standard commercial production (C) environments. These two environments represent the standards for biosecurity used in commercial breeding programs and the C environment represents normal commercial production conditions in Western countries. In both systems, birds were reared in large flocks under floor conditions. The data included BW records from 16 time-steps (TS) of selection that covered roughly 2.5 generations. A time-step involves selection and testing activities in the breeding programs that are carried out within a relatively short period of time. Birds that hatched at each TS were transferred to either the B or C environment for phenotype testing. Each full sib group was split between the B and C environments such that each bird will have full and half-sibs in both environments. Parents were selected from birds tested in the B environment only. In other words, parents did not have any phenotypic records in the C environment. In each TS, all the offspring of birds in the C environment were hatched at the same time while the offspring of birds in the B environment were hatched at several successive time points. Sires and dams of the offspring in each TS were from several previous TS.

BW of the broilers tested in the B environment was recorded once, at 6 weeks of age (BW6.B), for TS 1–10 and recorded once or twice, at 5 and 6 weeks of age (BW5.B and BW6.B), for TS 11–16. All birds in the B environment at TS 11–16 had BW5 records, but only 33% of those birds had BW6 records. The BW of the broilers tested in the C environment were recorded at 5 and 6 weeks of age (BW5.C and BW6.C) for TS 5–10 and only at 5 weeks of age (BW5.C) for TS 11–16. Data editing was performed as in Chapter 4 of Chu [26] by removing records of the birds with unidentified sex, missing dam age or duplicated records. Records of BW that were more than four standard deviation units from the mean were also removed for each of the four BW record types. In total, 0.04% of all BW records were removed. After data editing, 54,757 and 15,412 birds remained in the B and C environments, respectively, with respectively 61,589 and 23,569 BW records. The birds with BW records were from 319 sires and 1528 dams. The pedigree covered roughly 3.5 generations back from the youngest birds and included 70,174 birds.

A medium-density SNP chip with 55,792 SNPs was used for genotyping (Illumina, San Diego, CA, USA). For quality control, the missing rate for SNPs was set at < 0.05 and the call rate for birds at > 0.95. Also, SNPs with a minor allele frequency lower than 0.01 were removed. After quality control, 39,767 genotyped birds with 50,562 SNPs remained for constructing the genomic relationship matrix. All parents had genotype information. Although all the birds in the C environment were genotyped, after quality control, genotyping information of 41 birds in C was not used to construct the genomic relationship matrix. Only part of the birds in the B environment were genotyped.

### Statistical models

A preliminary study showed that male and female BW had different variances, but were highly correlated, which reflects scaling effects [26]. Modelling male and female BW as two traits led to convergence problems because of paramete at the edge of the parameter space. To model heterogeneous variances between male and female BW, standardization was applied to male and female BW, separately [26]. Male and female phenotypic records of the four BW traits were standardized to their corresponding phenotypic standard deviations that were estimated by the following univariate model:

1 $${\mathbf{y}} = {\mathbf{Xb}} + {\mathbf{Za}} + {\mathbf{Wc}} + {\mathbf{e}},$$

where **y** is a vector of male or female phenotypic records of BW at the original scale; **b** is a vector of fixed factors of hatch TS of the bird, TS of the parents, and dam age in classes of 1 week; **X**, **Z**, and **W** are incidence matrices; **a**, **c** and **e** are the vectors of the direct additive genetic effects, permanent environmental maternal effects and residuals, respectively. These random effects were assumed to be normally distributed: ${\mathbf{a}} \sim N\left[ {{\mathbf{0}},{\mathbf{A}}\sigma_{a}^{2} } \right]$, ${\mathbf{c}} \sim N\left[ {{\mathbf{0}},{\mathbf{I}}_{{\mathbf{d}}} \sigma_{c}^{2} } \right]$ and ${\mathbf{e}} \sim N\left[ {{\mathbf{0}},{\mathbf{I}}\sigma_{e}^{2} } \right]$, where **A** is the pedigree relationship matrix; **I**_**d**_ is the identity matrix for dams; **I** is the identity matrix for individual birds; $\sigma_{a}^{2}$, $\sigma_{c}^{2}$ and $\sigma_{e}^{2}$ are variances at the original scale of BW.

Standardization was applied, so that each phenotypic record was divided by the corresponding phenotypic standard deviation estimated from model (1). A multi-trait PBLUP model was used to estimate variance components and predict EBV from the standardized phenotypic records of BW in the B and C environments. The PBLUP model (2) includes eight traits but after transformation, the variances of genetic and maternal effects were equalized and correlations between sexes were equal to 1, so rank reduction was used for these effects whereas individual variances and covariances were estimated for the residual. The model was as follows:

2 $$\begin{array}{*{20}c} {\left[ {\begin{array}{*{20}c} {{\mathbf{y}}_{{{\mathbf{5B}}}}^{{{\mathbf{m}}0}} } \\ {{\mathbf{y}}_{{{\mathbf{5B}}}}^{{{\mathbf{f0}}}} } \\ \end{array} } \right] = } & {\left[ {\begin{array}{*{20}c} {{\mathbf{X}}_{{{\mathbf{5B}}}}^{{\mathbf{m}}} } & 0 \\ 0 & {{\mathbf{X}}_{{{\mathbf{5B}}}}^{{\mathbf{f}}} } \\ \end{array} } \right]\left[ {\begin{array}{*{20}c} {{\mathbf{b}}_{{{\mathbf{5B}}}}^{{\mathbf{m}}} } \\ {{\mathbf{b}}_{{{\mathbf{5B}}}}^{{\mathbf{f}}} } \\ \end{array} } \right] + } & {\left[ {\begin{array}{*{20}c} {{\mathbf{Z}}_{{{\mathbf{5B}}}}^{{\mathbf{m}}} } & 0 \\ 0 & {{\mathbf{Z}}_{{{\mathbf{5B}}}}^{{\mathbf{f}}} } \\ \end{array} } \right]{\mathbf{a}}_{{{\mathbf{5B}}}} + } & {\left[ {\begin{array}{*{20}c} {{\text{W}}_{{{\mathbf{5B}}}}^{{\mathbf{m}}} } & 0 \\ 0 & {{\text{W}}_{{{\mathbf{5B}}}}^{{\mathbf{f}}} } \\ \end{array} } \right]{\mathbf{c}}_{{{\mathbf{5B}}}} + } & {\left[ {\begin{array}{*{20}c} {{\mathbf{e}}_{{{\mathbf{5B}}}}^{{\mathbf{m}}} } \\ {{\mathbf{e}}_{{{\mathbf{5B}}}}^{{\mathbf{f}}} } \\ \end{array} } \right]} \\ {\left[ {\begin{array}{*{20}c} {{\mathbf{y}}_{{{\mathbf{6B}}}}^{{{\mathbf{m0}}}} } \\ {{\mathbf{y}}_{{{\mathbf{6B}}}}^{{{\mathbf{f0}}}} } \\ \end{array} } \right] = } & {\left[ {\begin{array}{*{20}c} {{\mathbf{X}}_{{{\mathbf{6B}}}}^{{\mathbf{m}}} } & 0 \\ 0 & {{\mathbf{X}}_{{{\mathbf{6B}}}}^{{\mathbf{f}}} } \\ \end{array} } \right]\left[ {\begin{array}{*{20}c} {{\mathbf{b}}_{{{\mathbf{6B}}}}^{{\mathbf{m}}} } \\ {{\mathbf{b}}_{{{\mathbf{6B}}}}^{{\mathbf{f}}} } \\ \end{array} } \right] + } & {\left[ {\begin{array}{*{20}c} {{\mathbf{Z}}_{{{\mathbf{6B}}}}^{{\mathbf{m}}} } & 0 \\ 0 & {{\mathbf{Z}}_{{{\mathbf{6B}}}}^{{\mathbf{f}}} } \\ \end{array} } \right]{\mathbf{a}}_{{{\mathbf{6B}}}} + } & {\left[ {\begin{array}{*{20}c} {{\text{W}}_{{{\mathbf{6B}}}}^{{\mathbf{m}}} } & 0 \\ 0 & {{\text{W}}_{{{\mathbf{6B}}}}^{{\mathbf{f}}} } \\ \end{array} } \right]{\mathbf{c}}_{{{\mathbf{6B}}}} + } & {\left[ {\begin{array}{*{20}c} {{\mathbf{e}}_{{{\mathbf{6B}}}}^{{\mathbf{m}}} } \\ {{\mathbf{e}}_{{{\mathbf{6B}}}}^{{\mathbf{f}}} } \\ \end{array} } \right]} \\ {\left[ {\begin{array}{*{20}c} {{\mathbf{y}}_{{{\mathbf{5C}}}}^{{{\mathbf{m0}}}} } \\ {{\mathbf{y}}_{{{\mathbf{5C}}}}^{{{\mathbf{f0}}}} } \\ \end{array} } \right] = } & {\left[ {\begin{array}{*{20}c} {{\mathbf{X}}_{{{\mathbf{5C}}}}^{{\mathbf{m}}} } & 0 \\ 0 & {{\mathbf{X}}_{{{\mathbf{5C}}}}^{{\mathbf{f}}} } \\ \end{array} } \right]\left[ {\begin{array}{*{20}c} {{\mathbf{b}}_{{{\mathbf{5C}}}}^{{\mathbf{m}}} } \\ {{\mathbf{b}}_{{{\mathbf{5C}}}}^{{\mathbf{f}}} } \\ \end{array} } \right] + } & {\left[ {\begin{array}{*{20}c} {{\mathbf{Z}}_{{{\mathbf{5C}}}}^{{\mathbf{m}}} } & 0 \\ 0 & {{\mathbf{Z}}_{{{\mathbf{5C}}}}^{{\mathbf{f}}} } \\ \end{array} } \right]{\mathbf{a}}_{{{\mathbf{5C}}}} + } & {\left[ {\begin{array}{*{20}c} {{\text{W}}_{{{\mathbf{5C}}}}^{{\mathbf{m}}} } & 0 \\ 0 & {{\text{W}}_{{{\mathbf{5C}}}}^{{\mathbf{f}}} } \\ \end{array} } \right]{\mathbf{c}}_{{{\mathbf{5C}}}} + } & {\left[ {\begin{array}{*{20}c} {{\mathbf{e}}_{{{\mathbf{5C}}}}^{{\mathbf{m}}} } \\ {{\mathbf{e}}_{{{\mathbf{5C}}}}^{{\mathbf{f}}} } \\ \end{array} } \right]} \\ {\left[ {\begin{array}{*{20}c} {{\mathbf{y}}_{{{\mathbf{6C}}}}^{{{\mathbf{m0}}}} } \\ {{\mathbf{y}}_{{{\mathbf{6C}}}}^{{{\mathbf{f0}}}} } \\ \end{array} } \right] = } & {\left[ {\begin{array}{*{20}c} {{\mathbf{X}}_{{{\mathbf{6C}}}}^{{\mathbf{m}}} } & 0 \\ 0 & {{\mathbf{X}}_{{{\mathbf{6C}}}}^{{\mathbf{f}}} } \\ \end{array} } \right]\left[ {\begin{array}{*{20}c} {{\mathbf{b}}_{{{\mathbf{6C}}}}^{{\mathbf{m}}} } \\ {{\mathbf{b}}_{{{\mathbf{6C}}}}^{{\mathbf{f}}} } \\ \end{array} } \right] + } & {\left[ {\begin{array}{*{20}c} {{\mathbf{Z}}_{{{\mathbf{6C}}}}^{{\mathbf{m}}} } & 0 \\ 0 & {{\mathbf{Z}}_{{{\mathbf{6C}}}}^{{\mathbf{f}}} } \\ \end{array} } \right]{\mathbf{a}}_{{{\mathbf{6C}}}} + } & {\left[ {\begin{array}{*{20}c} {{\text{W}}_{{{\mathbf{6C}}}}^{{\mathbf{m}}} } & 0 \\ 0 & {{\text{W}}_{{{\mathbf{6C}}}}^{{\mathbf{f}}} } \\ \end{array} } \right]{\mathbf{c}}_{{{\mathbf{6C}}}} + } & {\left[ {\begin{array}{*{20}c} {{\mathbf{e}}_{{{\mathbf{6C}}}}^{{\mathbf{m}}} } \\ {{\mathbf{e}}_{{{\mathbf{6C}}}}^{{\mathbf{f}}} } \\ \end{array} } \right]} \\ \end{array}$$

where ${\mathbf{y}}_{{5{\mathbf{B}}}}^{{{\mathbf{m}}0}}$, ${\mathbf{y}}_{{6{\mathbf{B}}}}^{{{\mathbf{m}}0}}$, ${\mathbf{y}}_{{5{\mathbf{C}}}}^{{{\mathbf{m}}0}}$ and ${\mathbf{y}}_{{6{\mathbf{C}}}}^{{{\mathbf{m}}0}}$ are the vectors of standardized phenotypic records of BW5.B, BW6.B, BW5.C, and BW6.C, respectively, for males; ${\mathbf{y}}_{{5{\mathbf{B}}}}^{{{\mathbf{f}}0}}$, ${\mathbf{y}}_{{6{\mathbf{B}}}}^{{{\mathbf{f}}0}}$, ${\mathbf{y}}_{{5{\mathbf{C}}}}^{{{\mathbf{f}}0}}$ and ${\mathbf{y}}_{{6{\mathbf{C}}}}^{{{\mathbf{f}}0}}$ are the vectors of standardized phenotypic records of BW5.B, BW6.B, BW5.C, and BW6.C, respectively, for females; ${\mathbf{b}}_{{5{\mathbf{B}}}}^{{\mathbf{m}}}$, ${\mathbf{b}}_{{5{\mathbf{B}}}}^{{\mathbf{f}}}$, ${\mathbf{b}}_{{6{\mathbf{B}}}}^{{\mathbf{m}}}$, ${\mathbf{b}}_{{6{\mathbf{B}}}}^{{\mathbf{f}}}$, ${\mathbf{b}}_{{5{\mathbf{C}}}}^{{\mathbf{m}}}$, ${\mathbf{b}}_{{5{\mathbf{C}}}}^{{\mathbf{f}}}$, ${\mathbf{b}}_{{6{\mathbf{C}}}}^{{\mathbf{m}}}$ and ${\mathbf{b}}_{{6{\mathbf{C}}}}^{{\mathbf{f}}}$ are the vectors of fixed factors as in the above model (1); ${\mathbf{X}}_{{5{\mathbf{B}}}}^{{\mathbf{m}}}$, ${\mathbf{X}}_{{5{\mathbf{B}}}}^{{\mathbf{f}}}$, ${\mathbf{X}}_{{6{\mathbf{B}}}}^{{\mathbf{m}}}$, ${\mathbf{X}}_{{6{\mathbf{B}}}}^{{\mathbf{f}}}$, ${\mathbf{X}}_{{5{\mathbf{C}}}}^{{\mathbf{m}}}$, ${\mathbf{X}}_{{5{\mathbf{C}}}}^{{\mathbf{f}}}$, ${\mathbf{X}}_{{6{\mathbf{C}}}}^{{\mathbf{m}}}$, ${\mathbf{X}}_{{6{\mathbf{C}}}}^{{\mathbf{f}}}$, ${\mathbf{Z}}_{{5{\mathbf{B}}}}^{{\mathbf{m}}}$, ${\mathbf{Z}}_{{5{\mathbf{B}}}}^{{\mathbf{f}}}$, ${\mathbf{Z}}_{{6{\mathbf{B}}}}^{{\mathbf{m}}}$, ${\mathbf{Z}}_{{6{\mathbf{B}}}}^{{\mathbf{f}}}$, ${\mathbf{Z}}_{{5{\mathbf{C}}}}^{{\mathbf{m}}}$, ${\mathbf{Z}}_{{5{\mathbf{C}}}}^{{\mathbf{f}}}$, ${\mathbf{Z}}_{{6{\mathbf{C}}}}^{{\mathbf{m}}}$, ${\mathbf{Z}}_{{6{\mathbf{C}}}}^{{\mathbf{f}}}$, ${\mathbf{W}}_{{5{\mathbf{B}}}}^{{\mathbf{m}}}$, ${\mathbf{W}}_{{5{\mathbf{B}}}}^{{\mathbf{f}}}$, ${\mathbf{W}}_{{6{\mathbf{B}}}}^{{\mathbf{m}}}$, ${\mathbf{W}}_{{6{\mathbf{B}}}}^{{\mathbf{f}}}$, ${\mathbf{W}}_{{5{\mathbf{C}}}}^{{\mathbf{m}}}$, ${\mathbf{W}}_{{5{\mathbf{C}}}}^{{\mathbf{f}}}$, ${\mathbf{W}}_{{6{\mathbf{C}}}}^{{\mathbf{m}}}$ and ${\mathbf{W}}_{{6{\mathbf{C}}}}^{{\mathbf{f}}}$ are incidence matrices; ${\mathbf{a}}_{{5{\mathbf{B}}}}$, ${\mathbf{a}}_{{6{\mathbf{B}}}}$, ${\mathbf{a}}_{{5{\mathbf{C}}}}$ and ${\mathbf{a}}_{{6{\mathbf{C}}}}$ are vectors of the direct additive genetic effects that were reduced ranks [27] for male and female traits: ${\mathbf{a}}_{{5{\mathbf{B}}}}$, ${\mathbf{a}}_{{6{\mathbf{B}}}}$, ${\mathbf{a}}_{{5{\mathbf{C}}}}$ and ${\mathbf{a}}_{{6{\mathbf{C}}}}$ $\sim {\mathbf{MVN}}\left[ {0 , {\mathbf{A}} \otimes {\mathbf{V}}_{{\mathbf{a}}}^{0} } \right]$, where ${\mathbf{V}}_{{\mathbf{a}}}^{0}$ is the 4 × 4 covariance matrix; ${\mathbf{A}}$ is the pedigree relationship matrix; ${\mathbf{MVN}}$ is the multivariate normal distribution; and ⊗ is the Kronecker product. ${\mathbf{c}}_{{5{\mathbf{B}}}}$, ${\mathbf{c}}_{{6{\mathbf{B}}}}$, ${\mathbf{c}}_{{5{\mathbf{C}}}}$ and ${\mathbf{c}}_{{6{\mathbf{C}}}}$ are the vectors of permanent environmental maternal effects that were reduced ranks [27] for male and female traits: ${\mathbf{c}}_{{5{\mathbf{B}}}}$, ${\mathbf{c}}_{{6{\mathbf{B}}}}$, ${\mathbf{c}}_{{5{\mathbf{C}}}}$ and ${\mathbf{c}}_{{6{\mathbf{C}}}} \sim {\mathbf{MVN}}\left[ {0 , {\mathbf{I}}_{{\mathbf{d}}} \otimes {\mathbf{V}}_{{\mathbf{c}}}^{0} } \right]$, where ${\mathbf{V}}_{{\mathbf{c}}}^{0}$ is the 4 × 4 covariance matrix; and ${\mathbf{I}}_{{\mathbf{d}}}$ is the identity matrix for dams. ${\mathbf{e}}_{{5{\mathbf{B}}}}^{{\mathbf{m}}}$, ${\mathbf{e}}_{{5{\mathbf{B}}}}^{{\mathbf{f}}}$,${\mathbf{e}}_{{6{\mathbf{B}}}}^{{\mathbf{m}}}$, ${\mathbf{e}}_{{6{\mathbf{B}}}}^{{\mathbf{f}}}$, ${\mathbf{e}}_{{5{\mathbf{C}}}}^{{\mathbf{m}}}$, ${\mathbf{e}}_{{5{\mathbf{C}}}}^{{\mathbf{f}}}$, ${\mathbf{e}}_{{6{\mathbf{C}}}}^{{\mathbf{m}}}$ and ${\mathbf{e}}_{{6{\mathbf{C}}}}^{{\mathbf{f}}}$ are the vectors of residuals $\sim {\mathbf{MVN}}\left[ {0 , {\mathbf{I}} \otimes {\mathbf{V}}_{{\mathbf{e}}}^{0} } \right]$, where ${\mathbf{V}}_{{\mathbf{e}}}^{0}$ is the 8 × 8 covariance matrix because heterogeneous variances between sexes were applied to the residuals in model (2); ${\mathbf{I}}$ is the identity matrix for individual birds. In the matrix, ${\mathbf{V}}_{{\mathbf{e}}}^{0}$, covariances between male and female traits, and between traits in B and C were zero. The covariance matrices ${\mathbf{V}}_{{\mathbf{a}}}^{0}$, ${\mathbf{V}}_{{\mathbf{c}}}^{0}$ and ${\mathbf{V}}_{{\mathbf{e}}}^{0}$ are at the standardized scale.

Variance components were estimated using the PBLUP multivariate model (2) for two reasons: (1) the selective genotyping applied to birds in the B environment might lead to overestimation and bias of variance components estimates with a ssGBLUP model [25]; and (2) the complexity of the models and large number of genotyped animals and non-genotyped animals in the pedigree would make ssGBLUP computationally demanding. The estimation of variance components and EBV prediction with the PBLUP models were carried out by using the REML module in the DMUAI procedure of the DMU package [28]. For the convergence of the model, the Frobenius norm of the update vector was set to less than 10^−5^ [28]. In addition, the PBLUP models used to estimate the variance components were re-run several times using different starting values to check that the global maximum likelihood of the model was reached. The model was converged, and no change in estimates was observed for different starting values.

The standardized variance components of the PBLUP model estimated from the REML module were used to calculate EBV for ssGBLUP multivariate models using the DMU5 procedure in the DMU package [28]. Because the use of genomic information led to a relatively dense relationship matrix, calculation of the sparse inverse of the LHS was not possible. Therefore, the DMU5 procedure was used to iteratively solve the mixed model equations with the preconditioned conjugate gradient method [28]. However, this procedure does not provide the prediction error variance of breeding values. The ssGBLUP models were identical to the PBLUP model (2), except that the pedigree relationship matrix ${\mathbf{A}}$ was replaced by a combined relationship matrix ${\mathbf{H}}$. Matrix ${\mathbf{H}}$ was constructed from the pedigree relationship matrix ${\mathbf{A}}$ and the genomic relationship matrix ${\mathbf{G}}$ with the weight value *ω* = 0.01 [8, 29] on the pedigree relationships. The genomic relationship matrix ${\mathbf{G}}$ was constructed based on SNP data, using method 1 from VanRaden [30].

### Cross-validation

Cross-validation was carried out to evaluate accuracy, bias and dispersion of EBV for genotyped and non-genotyped validation birds in the B environment. Validation was based on EBV estimated from the full and reduced datasets. The full dataset contained all the phenotypic records from TS 1–16, whereas the reduced dataset contained only phenotypic records from TS 1–12. The reduced dataset was a subset of the full dataset, from which records of 14,187 birds in the B environment and 5988 birds in the C environment at TS 13–16 were removed. The validation birds were individuals in the B environment at TS 13–16. Such a design was used to avoid having two or more generations of validation birds. The validation individuals can be genotyped birds or non-genotyped birds. Cross-validation measures were EBV statistics for validation birds that were estimated from the full and reduced datasets [23], i.e. the correlation ($$\rho_{f,r}$$) between $${\mathbf{EBV}}_{f}$$ and $${\mathbf{EBV}}_{r}$$, the difference ($$d_{f,r} )$$ in means between $${\mathbf{EBV}}_{f}$$ and $${\mathbf{EBV}}_{r}$$ and the regression slope ($$b_{f,r}$$) of $${\mathbf{EBV}}_{f}$$ on $${\mathbf{EBV}}_{r}$$, where $${\mathbf{EBV}}_{f}$$ and $${\mathbf{EBV}}_{r}$$ are vectors of EBV of the validation birds that are estimated from the full and reduced datasets, respectively. The statistics $$\rho_{f,r}$$, $$d_{f,r}$$ and $$b_{f,r}$$ are indicators of population accuracy, bias and dispersion of EBV, respectively [23]. Expectation of $$\rho_{f,r}$$ is $$\frac{{acc_{r} }}{{acc_{f} }}$$ [23], where $$acc_{r}$$ is the population accuracy of EBV defined as the correlation between the true breeding values and $${\mathbf{EBV}}_{r}$$; $$acc_{f}$$ is the population accuracy of EBV defined as the correlation between the true breeding values and $${\mathbf{EBV}}_{f}$$. Expectations of $$d_{f,r}$$ and $$b_{f,r}$$ are 0 and 1, respectively [23].

The two validation models were the PBLUP and ssGBLUP models. Standardized covariance components that were estimated from the PBLUP model using the full dataset were used to predict EBV in the PBLUP and ssGBLUP models using the full or reduced datasets. The vectors ${\mathbf{EBV}}_{f}$ and ${\mathbf{EBV}}_{r}$ were at the standardized scale. Scaling does not influence $\rho_{f,r}$ and $b_{f,r}$. However, scaling applied differently to records of each sex, which means that $d_{f,r}$ would have two values for males and females at the original scale. To compare models, $d_{f,r}$ was computed at the standardized scale. Standard errors of $\rho_{f,r}$, $d_{f,r}$ and $b_{f,r}$ were calculated using the formula in Appendix.

### Rescaling of parameters

Parameters that were estimated from the PBLUP model (2) were on the standardized scale for all BW traits. The estimates were re-scaled back to the original scale for male and female BW traits. Rescaling of (co)variance matrices used formula (3) to (5):

3 $${\mathbf{V}}_{\varvec{a}} = {\mathbf{T}}_{2} \left( {{\mathbf{T}}_{1} {\mathbf{V}}_{\varvec{a}}^{0} {\mathbf{T}}_{1}^{{\prime }} } \right) {\mathbf{T}}_{2}^{{\prime }} ,$$

4 $${\mathbf{V}}_{\varvec{c}} = {\mathbf{T}}_{2} \left( {{\mathbf{T}}_{1} {\mathbf{V}}_{\varvec{c}}^{0} {\mathbf{T}}_{1}^{{\prime }} } \right) {\mathbf{T}}_{2}^{{\prime }} ,$$

5 $${\mathbf{V}}_{\varvec{e}} = {\mathbf{T}}_{2} {\mathbf{V}}_{\varvec{e}}^{0} {\mathbf{T}}_{2}^{{\prime }} ,$$

where the matrices of direct additive genetic, permanent environmental maternal, residual and asymptotic covariances were ${\mathbf{V}}_{\varvec{a}}$, ${\mathbf{V}}_{\varvec{c}}$ and ${\mathbf{V}}_{\varvec{e}}$, respectively, at the original scale, and ${\mathbf{V}}_{\varvec{a}}^{0}$, ${\mathbf{V}}_{\varvec{c}}^{0}$ and ${\mathbf{V}}_{\varvec{e}}^{0}$, respectively, at the standardized scale. The transforming matrix ${\mathbf{T}}_{1}$ was:

$${\mathbf{T}}_{1} = \left[ {\begin{array}{*{20}c} 1 & 0 & 0 & 0 \\ 1 & 0 & 0 & 0 \\ 0 & 1 & 0 & 0 \\ 0 & 1 & 0 & 0 \\ 0 & 0 & 1 & 0 \\ 0 & 0 & 1 & 0 \\ 0 & 0 & 0 & 1 \\ 0 & 0 & 0 & 1 \\ \end{array} } \right].$$

Matrix ${\mathbf{T}}_{2}$ is an 8 × 8 matrix, where the off-diagonal elements are zero, the diagonal is vector of phenotypic standard deviations with trait orders: male BW5.B, female BW5.B, male BW6.B, female BW6.B, male BW5.C, female BW5.C, male BW6.C and female BW6.C. The phenotypic standard deviations of diagonals from matrix ${\mathbf{T}}_{2}$ were computed from the univariate model (1) for the corresponding traits. The asymptotic covariance matrix, which was used to compute approximate standard errors, was also transformed to the observed scale as in Fischer et al. [31].

## Results

The number of records, means, and standard deviations of BW records for broiler chicken raised in the B and C environments are in Table 1. The mean BW in the B environment was larger than that in the C environment for the same week and sex. However, the standard deviation of BW in the B environment was smaller than that in the C environment, and thus the coefficient of variation of BW in the B environment was lower. For example, coefficients of variation were 0.098 and 0.174 for male BW5 measured in the B and C environments, respectively.

**Table 1:** Descriptive statistics for body weight (BW) records of broiler chicken at 5 and 6 weeks of age for each sex raised in breeding (B) and commercial production (C) environments

BW at week	Sex	B environment			C environment		
		Number of records	Mean	Standard deviation	Number of records	Mean	Standard deviation
5	Male	10,117	2183	213	7455	1735	302
5	Female	10,801	1882	180	7922	1550	248
6	Male	18,651	2758	269	3975	2231	364
6	Female	22,020	2329	217	4217	1940	290

BW was measured in g

The standard deviation of BW records increased from 5 to 6 weeks of age and the mean BW increased by 500 to 600 g between weeks 5 and 6. As a result, the change in the coefficients of variation was relatively small. For example, the coefficient of variation for BW records of male birds in B environment was equal to 0.098 at both weeks 5 and 6 and those of male broilers in the C environment were equal to 0.174 and 0.163 at weeks 5 and 6, respectively.

Mean BW was greater in males than in females at the same age. The standard deviation of BW was also higher in males than in females, and thus the difference in coefficients of variation between sexes was relatively small. The relative difference in means of BW between males and females was larger for records in the B than the C environment. The relative difference in standard deviations between males and females was smaller for records in the B than the C environment.

Table 1 does not show separated statistics for genotyped and non-genotyped birds in the B environment. However, genotyped birds in the B environment had larger means and lower standard deviations for BW records than the non-genotyped birds at the corresponding weeks of age. For example, the mean and standard deviation of BW6 of males were equal to 2814 and 248 for genotyped birds, while they were 2707 and 278 for non-genotyped birds, respectively.

Additive genetic variances, heritabilities and permanent environmental maternal effects of traits measured in the B and C environments for male and female BW5-6 were estimated from the PBLUP model (2) and are in Table 2. Genetic variances of the traits measured in the C environment were considerably higher than those measured in the B environment. The relative differences in variances between traits measured in the B and C environments for male BW5, female BW5, male BW6 and female BW6 were equal to 2.39, 2.29, 2.29 and 2.04, respectively.

**Table 2:** Estimates of direct additive genetic variance ($\sigma_{a}^{2}$), heritability ($h^{2}$) and permanent environmental maternal effect ($c^{2}$) estimated from male and female body weight (BW) at weeks 5 and 6 in breeding (B) and commercial production (C) environments

BW at week	Sex	B environment			C environment		
		$\sigma_{a}^{2}$	$h^{2}$	$c^{2}$	$\sigma_{a}^{2}$	$h^{2}$	$c^{2}$
5	Male	10,454	0.274	0.033	24,984	0.358	0.037
5	Female	7614	0.278	0.033	17,469	0.366	0.038
6	Male	17,301	0.301	0.034	39,544	0.312	0.028
6	Female	11,651	0.298	0.034	23,831	0.305	0.027
Range of SE			0.022–0.024	0.007–0.008		0.033–0.037	0.011–0.013

*SE* standard errors

Estimates of the heritability of traits measured in the C environment tended to be higher than those measured in the B environment. Heritabilities of BW5-6 ranged from 0.27 to 0.30 and from 0.31 to 0.37 for the same traits measured in the B and C environments, respectively. With age increasing from 5 to 6 weeks, heritability of the traits measured in C decreased, whereas that of the traits measured in B tended to increase. The ratio of the permanent environmental maternal variance to the total phenotypic variance for the traits measured in C decreased from week 5 to 6, whereas that for the traits measured in B tended to increase.

The genetic variance for BW was considerably larger in males than in females when considering the traits in the same environment and at the same week of age. The absolute and relative differences in variances between male and female traits were larger for traits measured in C than in B. However, the differences in heritabilities and permanent environmental maternal effects were mostly negligible between male and female traits measured in the same environment and at the same week of age.

Estimates of variances, heritabilities and permanent environmental maternal effects differed between male and female traits because the PBLUP model uses heterogeneous residual variances and different scaling for sexes. However, genetic correlations and permanent environmental maternal correlations between sexes were assumed to be 1 for the same traits. Therefore, a single correlation estimate was obtained for the different sexes combined. Table 3 shows the estimated values from the PBLUP model of the genetic correlations and permanent environmental maternal correlations between BW measured in B and C at weeks 5 and 6.

**Table 3:** Genetic correlations (above diagonal), permanent environmental maternal correlations (below diagonal) of body weight (BW) of broiler chicken reared in breeding (B) and commercial production (C) environments at 5 and 6 weeks of age

BW at week	Environment	B		C	
		5	6	5	6
5	B	1	0.956	0.535	0.490
6		0.953	1	0.497	0.479
5	C	0.690	0.589	1	0.989
6		0.723	0.628	0.999	1

Standard errors: genetic correlations ± 0.010–0.064; permanent environmental maternal correlations ± 0.027–0.155

The genetic correlations between BW measured in B and C ranged from 0.48 to 0.54. The genetic correlation of BW between the B and C environments tended to decrease as week of age increased from 5 to 6. Genetic correlations of 0.54 between BW5.B and BW5.C and 0.48 between male BW6.B and BW6.C were obtained. Genetic variances in each environment were larger for BW6 than BW5. The correlation between BW5 and BW6 tended to be higher when measured in C (0.99) than when measured in B (0.96).

The permanent environmental maternal correlations between traits measured in B and C ranged from 0.59 to 0.72. The permanent environmental maternal correlations between BW at 5 and 6 weeks and BW measured in B and C followed similar trends as the genetic correlations. For example, the environmental correlations between environments B and C were lower for BW6 than for BW5. Permanent environmental maternal correlations between BW5 and BW6 in the same environment were very high but still a bit higher for traits measured in C than in B.

The $\rho_{f,r}$, $b_{f,r}$ and $d_{f,r}$ statistics for the PBLUP and ssGBLUP models were calculated based on EBV estimated from the full and reduced datasets (Table 4). These cross-validation measures were computed separately for genotyped and non-genotyped validation birds for EBV of all BW traits. The use of ssGBLUP increased $\rho_{f,r}$ for all validation birds, and decreased $d_{f,r}$ for genotyped birds compared to the use of PBLUP.

**Table 4:** Cross-validation measures for genotyped and non-genotyped validation birds in the B environment using pedigree-based BLUP (PBLUP) and single-step GBLUP (ssGBLUP) models

Model	Validated traits	Genotyped validation birds			Non-genotyped validation birds		
		$\rho_{f,r}$	$b_{f,r}$	$d_{f,r}$	$\rho_{f,r}$	$b_{f,r}$	$d_{f,r}$
PBLUP	BW5.B	0.509	0.898	0.106	0.588	0.874	− 0.081
	BW6.B	0.457	0.858	0.129	0.570	0.849	− 0.083
	BW5.C	0.616	1.055	0.088	0.678	1.026	− 0.052
	BW6.C	0.591	1.024	0.084	0.670	1.014	− 0.048
Range of SE		0.013–0.015	0.023–0.028	0.004–0.006	0.007–0.008	0.011–0.012	0.002–0.003
ssGBLUP	BW5.B	0.767	0.913	0.026	0.625	0.841	− 0.095
	BW6.B	0.791	0.946	0.043	0.653	0.867	− 0.091
	BW5.C	0.811	0.937	− 0.001	0.758	0.951	− 0.076
	BW6.C	0.813	0.939	0.000	0.770	0.965	− 0.069
Range of SE		0.010–0.011	0.011–0.013	0.003–0.004	0.006–0.008	0.008–0.010	0.002–0.003

For the validation birds, the traits were body weight (BW) at 5 and 6 weeks of age measured in the breeding (B) and commercial production (C) environments

$\rho_{f,r}$ is the correlation between ${\mathbf{EBV}}_{f}$ and ${\mathbf{EBV}}_{r}$; $b_{f,r}$ is the regression slope of ${\mathbf{EBV}}_{f}$ on ${\mathbf{EBV}}_{r}$; and $d_{f,r}$ is the mean of ${\mathbf{EBV}}_{f}$  −  ${\mathbf{EBV}}_{r}$; ${\mathbf{EBV}}_{f}$ and ${\mathbf{EBV}}_{r}$ are vectors of breeding values of the validation birds measured in B and estimated from the full and reduced datasets, respectively

*SE* standard errors

With the PBLUP model, $\rho_{f,r}$ and $d_{f,r}$ for the genotyped birds were respectively lower and higher than for the non-genotyped birds. For BW traits measured in B, $b_{f,r}$ for the genotyped birds was closer to 1 than that for the non-genotyped birds. In contrast, for BW traits measured in C, $b_{f,r}$ for the non-genotyped birds was closer to 1. With the PBLUP model, EBV of BW traits measured in B were deflated, but EBV of BW traits measured in C were inflated.

With the ssGBLUP model, $\rho_{f,r}$ for the genotyped birds was higher than for the non-genotyped birds. When the model was changed from PBLUP to ssGBLUP, $\rho_{f,r}$ increased for both genotyped birds and non-genotyped birds. However, the increase in $\rho_{f,r}$ was much larger for genotyped birds than for non-genotyped birds. For example, the relative increase in $\rho_{f,r}$ ranged from 31.7 to 73.1% for the genotyped birds and from 6.3 to 14.9% for the non-genotyped birds. $d_{f,r}$ for the genotyped birds was lower with the ssGBLUP than with the PBLUP model, whereas $d_{f,r}$ for the non-genotyped birds was higher with the ssGBLUP than with the PBLUP model. When the model was changed from PBLUP to ssGBLUP, $b_{f,r}$ for the genotyped birds increased for traits measured in B and decreased for traits measured in C, but $b_{f,r}$ for the non-genotyped birds decreased for traits measured in B and increased for traits measured in C. With the ssGBLUP model, EBV were deflated for all traits.

Regardless of the model used, $\rho_{f,r}$ and $d_{f,r}$ were respectively lower and higher for traits measured in B than for traits measured in C. For example, with PBLUP, $\rho_{f,r}$ for non-genotyped birds was 0.59 for BW5.B and 0.68 for BW5.C, and $d_{f,r}$ for non-genotyped birds was − 0.081 for BW5.B and − 0.052 for BW5.C.

## Discussion

Genetic parameters for male and female BW at 5 and 6 weeks of age raised in bio-secure breeding (B) and commercial production (C) environments were estimated using a PBLUP multivariate model. A multivariate ssGBLUP model was used to predict EBV of BW measured in B and C environments. Cross-validations were carried out to assess population accuracy, bias and dispersion of EBV predictions of traits measured in C for genotyped and non-genotyped birds in the B environment when the PBLUP and ssGBLUP models were used.

### Genotype-by-environment interactions

The difference between the B and C environments is mainly determined by hygiene conditions. The strict bio-secure conditions of the B environment are regulated to prevent worldwide spread of diseases to production farms and to avoid the risk of losing the purebred lines [1]. In contrast, the less strict hygiene conditions of the C environment can result in a higher incidence of diseases. For example, purebred birds in large commercial breeding companies are typically disease-free from salmonella, mycoplasma, leucosis, avian influenza and Newcastle disease [32]. However, these diseases remain chronic problems in many commercial poultry flocks [33–35]. The difference between the B and C environments may also be related to diet and litter management. Commonly, wood shavings are used and are loose, dry, free-flowing in the B environment [32]. The better litter management in the B environment results in a substantially lower incidence of food-pad dermatitis compared to the C environment [1]. In our study, several indications of G × E interactions due to the differences between the B and C environments were found such as a change in average performance, re-rankings, heterogeneous variances and different heritabilities for the traits measured in the B and C environments. At the same age, the mean BW measured in B was larger than that measured in C, but the standard deviation of BW measured in B was lower than that measured in C. This result agrees with that of Kapell et al. [1] who reported larger means and lower standard deviations for BW measured in B than in C for four different purebred lines of broiler chicken raised in the B and C environments. In N’Dri et al. [3], slow-growing broiler chicken also had significantly higher BW performance when raised in the B environment compared to the C environment.

In our study, estimates of the genetic correlation between BW measured in B and C ranged from 0.479 to 0.535. Statistically, re-ranking refers to genetic correlations different from 1, but in practice, re-ranking is commonly considered important in breeding programs when the correlation is lower than 0.8 [36]. The genetic correlation in our study refers to a significant re-ranking of birds in the B and C environments. Such a significant re-ranking was also observed by Kapell et al. [1] with genetic correlations between B and C environments of 0.46, 0.54, 0.56 and 0.69 for their four studied lines, respectively. In N’Dri et al. [3], the genetic correlations ranged from 0.74 to 0.76. In Lwelamira [4], the genetic correlation between BW of indigenous chicken measured in breeding station and village environments was around 0.75–0.76. However, the estimates in Lwelamira [4] and N’Dri et al. [3] might be slightly overestimated because no permanent environmental maternal effect was included in the model and the datasets comprised a small number of records for only one generation.

With the more challenging C environment, we did expect larger variances for traits measured in C than for those measured in B, but we also obtained higher heritabilities for traits measured in C than for the same ones measured in B. Kapell et al. [1] found that, in three of their four lines, the heritability of BW5 measured in C (0.32–0.34) was lower than that measured in B (0.36–0.40), while in the remaining line, the heritability of BW5 measured in C (0.36) was higher than that measured in B (0.32). N’Dri et al. [3] reported heritabilities of traits measured in C that ranged from 0.54 to 0.56, whereas for those measured in B the heritability was 0.56. In addition, as week of age increased from 5 to 6, the heritability of traits measured in B tended to increase, whereas that of traits measured in C decreased. There is no clear explanation for these opposite trends. As age increased from 5 to 6 weeks, the genetic correlation between traits measured in B and C decreased. To our knowledge, no such trend in the genetic correlation between traits measured in B and C environments with increasing age has been previously reported in poultry.

The production environment also influenced the maternal environmental effects. Permanent environmental maternal correlations between BW measured in B and C were significantly lower than 1. The permanent environmental maternal effect of traits measured in C became smaller as age increased from 5 to 6 weeks. In contrast, the permanent environmental maternal effect of traits measured in B tended to increase as age increased from 5 to 6 weeks. In previous studies in poultry, a reduction in the permanent environment maternal effect on BW with increasing age was found [37–40]. However, some studies [41, 42] also show increasing trends around the age of 5 to 6 weeks, i.e. Begli et al. [41] showed that the ratio of the permanent environmental maternal variance to the total phenotypic variance for BW increased slightly from 0.10 at week 2 to 0.12 at week 6, and then decreased to 0.07 at week 10 and Mebratie et al. [42] showed that the maternal effect on BW of broiler chicken raised in B tended to increase as the age of the birds increased from *t*-7, to *t*-4 to *t* days.

The effects of the production environment on age-by-genotype interactions were not clear, but the genetic correlation between BW5 and BW6 tended to be lower for traits measured in B than in C. The effects of the production environment on sex-by-genotype interactions were mainly related to the relative difference in variances between male and female BW traits because the genetic correlation between male and female BW within each environment was assumed to be 1 in the multivariate model (2). This assumption was based on our preliminary results that the sex-by-genotype interaction only led to the scaling effect between male and female BW.

Based on the strong G × E interaction found in our study, selection for performance under commercial conditions will greatly increase response to selection for BW in broiler breeding programs when birds are phenotype-tested in both B and C environments [2, 43]. In contrast to the breeding programs that test birds in the B environment only, the breeding programs that test birds in both environments can exploit the re-ranking of birds in B and C, the larger genetic variances of traits measured in C (than those measured in B) and the higher heritability of traits measured in C (than those measured in B). Chu et al. [2] showed that with a genetic correlation between traits measured in B and C of 0.5, the scheme that placed 70 and 30% birds in the B and C environments, respectively, for phenotype testing, had substantially larger genetic gains than the scheme that had all birds tested in the B environment.

### Benefits of genomic information for the prediction of breeding values

An increase in population accuracy of EBV for genotyped birds has been shown in many studies [7–9, 14, 15, 22, 29, 44–46], in which population accuracy was assessed by the correlation between EBV and corrected phenotypes. Simulation studies have also shown that correlations between true breeding values and genomic EBV of genotyped individuals were significantly higher with ssGBLUP than with PBLUP models [7–10]. Applications of ssGBLUP to improve accuracy of selection have been well-documented in studies on chicken [11–15], cattle [16–19] and pig [10, 20–22]. However, the extent of the benefits from using genomic information for the accuracy of prediction has not been reported for breeding programs where sib-testing is used because of G × E interactions. In these breeding programs, the ultimate goal is to increase the genetic gains in the performance of traits in C for selection candidates that were raised in B, thus only the accuracy of EBV of BW traits measured in C is relevant. Our discussion focusses primarily on the accuracy of EBV of traits measured in C for the selection candidate birds in B that do not have own records for the traits measured in C.

The $\rho_{f,r}$, $d_{f,r}$ and $b_{f,r}$ statistics were indicators of population accuracy, bias and dispersion of EBV, respectively [23]. The $\rho_{f,r}$ statistic is a ratio of accuracies and prediction of EBV is expected to be more accurate when $\rho_{f,r}$ is higher with $\rho_{f,r}$ expected to be always lower than 1. Prediction of EBV is more biased as $d_{f,r}$ deviates more from 0. EBV would be more inflated or deflated when $b_{f,r}$ deviates more from 1. The use of combined pedigree and genomic information in ssGBLUP increased substantially the population accuracy of EBV for genotyped validation birds compared to the use of pedigree data only in PBLUP. Genomic information explains the relationships between individuals better than pedigree information. Thus, genomic information can be beneficial for an efficient “flow” of information from C to validation birds in B in several ways. With pedigree information, only up to 50% of the total genetic variance of the traits measured in C is exploited for the prediction of EBV of the candidates in a G × E sib-test. With genomic information, this percentage can be higher because the realized genomic relationships between full-sib individuals can range from 0.27 to 0.70 for broilers [47]. The prediction of PBLUP for validation birds typically treats phenotypic performances of their full-sibs in C as an average information whereas phenotypic performances of the full-sibs in C are treated individually in genomic prediction of EBV. In addition, information from related, more distantly related and even unrelated animals can be exploited in genomic prediction when genomic markers are in linkage disequilibrium with genotypes at causal loci [48]. The Mendelian sampling terms are better exploited with genomic information than with pedigree information [5, 48, 49], and thus accuracy of prediction from genomic information increases compared to the accuracy from pedigree information.

Most studies [7–9, 14, 15, 22, 29, 44–46] reported the regression slope of corrected phenotypes on EBV as bias, and showed an improvement in bias with genotyped animals. However, the methodology used for cross-validation in those studies is different from that in our study regarding e.g. validation animals and definition of bias. In our study, when the model was changed from PBLUP to ssGBLUP, dispersion or the regression slope of ${\mathbf{EBV}}_{f}$ on ${\mathbf{EBV}}_{r}$ for genotyped birds was improved for traits measured in B, but not for traits measured in C, and bias or difference in means between ${\mathbf{EBV}}_{f}$ and ${\mathbf{EBV}}_{r}$ for genotyped birds was improved for traits measured in B and C. In the literature, an increase in accuracy of EBV for non-genotyped individuals has also been reported when changing from PBLUP to ssGBLUP [18, 20–22], but the bias of prediction of these birds increased [18, 20, 21]. The cross-validation in these studies was based on corrected phenotypes and EBV. Nonetheless, these results are in agreement with our findings that show that accuracy of EBV of non-genotyped birds increased and bias of the prediction also increased.

Validation groups of genotyped and non-genotyped birds had different population accuracies of EBV even with the PBLUP model, which might be related to a non-random distribution of validation birds into the groups. In our dataset, the mean of BW measured in B was higher for genotyped birds than for non-genotyped birds, but the standard deviation of the records in B was lower for genotyped birds than for non-genotyped birds. All parents had genotyping information. This indicated that a selective genotyping strategy was applied for birds raised in B. Within the validation birds, the genotyped birds that are from the top of the distribution have higher relationships between individuals than the non-genotyped birds. From the numerator relationship matrix that was calculated from all animals in the pedigree, the average additive genetic relationship was 0.024 between validation genotyped birds and 0.019 between validation non-genotyped birds. The higher average relationship between genotyped birds results in less variation in EBV estimated from PBLUP, and thus lower population accuracy [50] for genotyped birds.

## Methodology

When genomic information was used and evaluated in a G × E sib-testing breeding program for broilers, our study was faced with several challenges. The estimation of variance components was challenging with such a large number of genotyped birds, computation-demanding multi-trait models, and selective genotyping. In addition, it was not clear which cross-validation strategies to use in the situation where selection candidates reside in B and do not have performance records in C. Cross-validation is commonly based on the correlation between phenotypes that are corrected for fixed effects from the PBLUP model using the full dataset and EBV that are estimated from PBLUP or ssGBLUP model using the reduced dataset [22]. However, corrected phenotypes for traits measured in C were not available for validation birds in B. To deal with these challenges, we used the PBLUP model to estimate variance components. The EBV statistics that were estimated from the full and reduced data for validation birds in B were used for validation measures e.g. indicators of population accuracy, bias and dispersion of EBV following the derivation of Legarra and Reverter [23].

Strictly, the $\rho_{f,r}$ statistic is not population accuracy, but a ratio of accuracies which is a direct indicator of population accuracy [23]. It describes the increase in population accuracy of EBV from reduced data to full data. We observed higher values of $\rho_{f,r}$ for traits measured in B than for those measured in C, but these values are not comparable for different traits. Without realizing that $\rho_{f,r}$ is a ratio, Putz et al. [10] reported a poor performance of this statistic for estimating accuracy of EBV. To estimate population accuracy from $\rho_{f,r}$, we need prediction error variances and covariances, and genetic variance at equilibrium in a population under selection [23]. However, instead of transforming it to population accuracy, $\rho_{f,r}$ can be used directly for comparisons of accuracy between competing statistical models or between subsets of animals in a population. Analytical properties of $\rho_{f,r}$, $d_{f,r}$ and $b_{f,r}$ were presented in Legarra and Reverter [23]. Using data of a simulated population and empirical data of a Brahman beef cattle population, they showed that there was very good agreement between the commonly used cross-validation that is based on corrected phenotypes and EBV estimated from a reduced dataset and the new cross-validation that is based on EBV estimated from reduced and full datasets.

The $\rho_{f,r}$ statistic is an indicator of population accuracy, not an indicator of individual accuracy [23]. Individual accuracy or model-based accuracy that could be obtained from prediction error variances was not used for model comparisons in our study, because it was not possible to obtain prediction error variances for the ssGBLUP model. In addition, individual accuracy reflects “the credibility of an individual EBV” or “a measure of the standard error of prediction of an individual EBV” [51]. Population accuracy reflects “the correlation between true breeding values and EBV among the candidates for selection, which is a property of a population, not of an individual” [51]. For example, when only parent average is known, individual accuracy of full-sibs is up to 0.71. However, the predicted differences between full-sibs have zero accuracy, or population accuracy among full-sibs is zero because full-sibs have the same parent average. Individual accuracy should be used for individual decisions, but population accuracy should be used for the choice of model and the assessment of genetic gain [23].

Sex-by-genotype interaction has been investigated for different traits in broiler chicken [42, 52, 53], other poultry [54, 55], pig and cattle [52, 56]. These studies showed that variances between sexes differed by a factor of 2 or more, but very little re-ranking between sexes was found with genetic correlations between male and female traits higher than 0.85. With a high genetic correlation, the implementation of a bivariate model that treats each sex as different traits could encounter convergence problems in the estimation of variance components. However, when the existence of heterogeneous variance for sexes was not accounted for in the model, a serious re-ranking could occur and thus lead to reduced response to selection [57]. Failure to account for different variances between sexes could also lead to bias in variance components and predicted breeding values [58]. Therefore, a relatively simple standardization was applied to male and female records to model heterogeneous variances between sexes without affecting convergence of the model. This standardization was also applied in Chapter 4 of Chu [26].

The data on BW5-6 of the birds measured in C used in the current study is the same as in Chapter 4 of Chu [26], but the models differed. Permanent environmental maternal effects in our study were higher than those reported by Chu [26]. The multivariate model in Chu [26] used BW1-2 and weekly weight gains from weeks 2 to 6 to model BW1-6, and this linear transformation led to the same inferences, but had much better convergence properties. However, because of the trade-off between the complexity of the model and convergence challenge, permanent environmental maternal effects were not included in the model for weekly weight gain from weeks 5 to 6. Therefore, in Chu [26] the direct additive genetic effects might be overestimated and the maternal effects underestimated. The model used in the current study was less complex since it included only BW at 5 and 6 weeks. In addition, to facilitate convergence, this study had slightly lower convergence criteria than the recommended value of 10^−6^ to 10^−5^ [28] for the Frobenius norms of the update vector and the gradient vector. Therefore, to ensure that the global maximum likelihood of the model was reached, the PBLUP models to estimate variance components were re-run several times with different starting values.

## Conclusions

Genetic parameters were estimated for male and female body weight (BW) at 5 and 6 weeks of chicken that were raised in breeding bio-secure (B) and production commercial environments (C). Several indications of interaction between genotype and testing environment (B and C) were found including different average performances, correlations significantly lower than 1, heterogeneous variances and different heritabilities for traits measured in B and C. In addition, genomic information on birds raised in both B and C was used for prediction of EBV of birds in B for BW traits measured in the B and C environments. To evaluate the prediction of EBV, cross-validation statistics of EBV were estimated, namely population accuracy, bias and dispersion of EBV from reduced and full datasets. We found that the use of combined pedigree and genomic information in ssGBLUP substantially increased population accuracy of EBV for genotyped birds compared to the use of only pedigree data in PBLUP. Accuracy of EBV also increased for non-genotyped birds, but bias of EBV prediction increased for non-genotyped birds. In summary, the differences between the B and C environments result in a strong G × E interaction with genetic correlations ranging from 0.479 to 0.535 between BW traits of broilers measured in the B and C environments. In order to ensure maximum genetic gain under commercial conditions, breeding programs should establish recording systems under commercial conditions to provide their customers with genotypes that are well adapted to the commercial environment. Compared to the use of pedigree data only, the use of combined pedigree and genomic information increases population accuracy of EBV substantially for genotyped birds that are raised in the B environment.

## Data Availability

The data that support the findings of this study are available on request from the corresponding author with permission from Cobb-Vantress.

## Appendix

Standard errors of the $\rho_{f,r}$, $d_{f,r}$ and $b_{f,r}$ statistics were calculated. A linear model for the vector ${\mathbf{EBV}}_{f}$ (called $\hat{u}_{{f_{i} }}$ in scalar notation) on the explanatory variable of vector ${\mathbf{EBV}}_{r}$ (called $\hat{u}_{{r_{i} }}$ in scalar notation) was assumed:

$$\hat{u}_{{f_{i} }} = \upalpha + b_{f,r} \hat{u}_{{r_{i} }} + \varepsilon_{i} ,$$

where $i$= 1, 2, …, $n$; $n$ is the length of vector ${\mathbf{EBV}}_{f}$ or ${\mathbf{EBV}}_{r}$; $\upalpha$ is the constant intercept; $b_{f,r}$ is the regression slope; and $\varepsilon_{i}$ is the residual term.

The standard error of the estimated regression slope ($\widehat{{b_{f,r} }}$) is:

$$SE\left( {\widehat{{b_{f,r} }}} \right) = \sqrt {\frac{{\frac{1}{n - 2}\mathop \sum \nolimits_{i = 1}^{n} \hat{\varepsilon }_{i}^{2} }}{{\mathop \sum \nolimits_{i = 1}^{n} \left( {\hat{u}_{{r_{i} }} - \overline{{\hat{u}_{r} }} } \right)^{2} }}} .$$

The standard error of the estimated correlation ($\widehat{{\rho_{f,r} }}$) between $\hat{u}_{{f_{i} }}$ and $\hat{u}_{{r_{i} }}$ is:

$$SE\left( {\widehat{{\rho_{f,r} }}} \right) = \frac{{1 - \widehat{{\rho_{f,r} }}^{2} }}{{\sqrt {n - 2} }}.$$

The standard error of the difference ($\widehat{{d_{f,r} }}$) between $\hat{u}_{{f_{i} }}$ and $\hat{u}_{{r_{i} }}$ is:

$$SE\left( {\widehat{{d_{f,r} }}} \right) = \frac{{\sqrt {\mathop \sum \nolimits_{i = 1}^{n} \left( {\hat{u}_{{f_{i} }} - \hat{u}_{{r_{i} }} - \left( {\overline {\widehat{{u_{f}} }} - \overline {\widehat{{u_{r} }}}} \right)} \right)^{2} } }}{n}.$$

## References

[1] Kapell DN, Hill WG, Neeteson AM, McAdam J, Koerhuis AN, Avendano S (2012). Genetic parameters of foot-pad dermatitis and body weight in purebred broiler lines in 2 contrasting environments. Poult Sci.

[2] Chu TT, Alemu SW, Norberg E, Sørensen AC, Henshall J, Hawken R (2018). Benefits of testing in both bio-secure and production environments in genomic selection breeding programs for commercial broiler chicken. Genet Sel Evol..

[3] N’Dri AL, Sellier N, Tixier-Boichard M, Beaumont C, Mignon-Grasteau S (2007). Genotype by environment interactions in relation to growth traits in slow growing chickens. Genet Sel Evol..

[4] Lwelamira J (2012). Genotype-environment (GXE) interaction for body weights for Kuchi chicken ecotype of Tanzania reared under intensive and extensive management. Glob J Med Res.

[5] Hayes BJ, Visscher PM, Goddard ME (2009). Increased accuracy of artificial selection by using the realized relationship matrix. Genet Res.

[6] Meuwissen THE, Hayes BJ, Goddard ME (2001). Prediction of total genetic value using genome-wide dense marker maps. Genetics.

[7] Andonov S, Lourenco DAL, Fragomeni BO, Masuda Y, Pocrnic I, Tsuruta S (2017). Accuracy of breeding values in small genotyped populations using different sources of external information—a simulation study. J Dairy Sci.

[8] Christensen OF, Lund MS (2010). Genomic prediction when some animals are not genotyped. Genet Sel Evol..

[9] Lourenco DA, Misztal I, Wang H, Aguilar I, Tsuruta S, Bertrand JK (2013). Prediction accuracy for a simulated maternally affected trait of beef cattle using different genomic evaluation models. J Anim Sci.

[10] Putz AM, Tiezzi F, Maltecca C, Gray KA, Knauer MT (2018). A comparison of accuracy validation methods for genomic and pedigree-based predictions of swine litter size traits using Large White and simulated data. J Anim Breed Genet.

[11] Wolc A, Stricker C, Arango J, Settar P, Fulton JE, O’Sullivan NP (2011). Breeding value prediction for production traits in layer chickens using pedigree or genomic relationships in a reduced animal model. Genet Sel Evol..

[12] Alemu SW, Calus MPL, Muir WM, Peeters K, Vereijken A, Bijma P (2016). Genomic prediction of survival time in a population of brown laying hens showing cannibalistic behavior. Genet Sel Evol..

[13] Momen M, Mehrgardi AA, Sheikhy A, Esmailizadeh A, Fozi MA, Kranis A (2017). A predictive assessment of genetic correlations between traits in chickens using markers. Genet Sel Evol..

[14] Chen CY, Misztal I, Aguilar I, Tsuruta S, Meuwissen THE, Aggrey S (2011). Genome-wide marker-assisted selection combining all pedigree phenotypic information with genotypic data in one step: an example using broiler chickens. J Anim Sci.

[15] Chen CY, Misztal I, Aguilar I, Legarra A, Muir WM (2011). Effect of different genomic relationship matrices on accuracy and scale. J Anim Sci.

[16] Lourenco DA, Tsuruta S, Fragomeni BO, Masuda Y, Aguilar I, Legarra A (2015). Genetic evaluation using single-step genomic best linear unbiased predictor in American Angus. J Anim Sci.

[17] Li X, Lund MS, Zhang Q, Costa CN, Ducrocq V, Su G (2016). Short communication: Improving accuracy of predicting breeding values in Brazilian Holstein population by adding data from Nordic and French Holstein populations. J Dairy Sci.

[18] Gao H, Koivula M, Jensen J, Strandén I, Madsen P, Pitkänen T (2018). Short communication: genomic prediction using different single-step methods in the Finnish red dairy cattle population. J Dairy Sci.

[19] Lee J, Cheng H, Garrick D, Golden B, Dekkers J, Park K (2017). Comparison of alternative approaches to single-trait genomic prediction using genotyped and non-genotyped Hanwoo beef cattle. Genet Sel Evol..

[20] Xiang T, Nielsen B, Su G, Legarra A, Christensen OF (2016). Application of single-step genomic evaluation for crossbred performance in pig. J Anim Sci.

[21] Guo X, Christensen OF, Ostersen T, Wang Y, Lund MS, Su G (2015). Improving genetic evaluation of litter size and piglet mortality for both genotyped and nongenotyped individuals using a single-step method. J Anim Sci.

[22] Christensen OF, Madsen P, Nielsen B, Ostersen T, Su G (2012). Single-step methods for genomic evaluation in pigs. Animal..

[23] Legarra A, Reverter A (2018). Semi-parametric estimates of population accuracy and bias of predictions of breeding values and future phenotypes using the LR method. Genet Sel Evol..

[24] Boligon AA, Long N, Albuquerque LG, Weigel KA, Gianola D, Rosa GJM (2012). Comparison of selective genotyping strategies for prediction of breeding values in a population undergoing selection. J Anim Sci.

[25] Cesarani A, Pocrnic I, Macciotta NPP, Fragomeni BO, Misztal I, Lourenco DAL (2019). Bias in heritability estimates from genomic restricted maximum likelihood methods under different genotyping strategies. J Anim Breed Genet.

[26] Chu TT. Genotype by environment interaction in poultry breeding programs. PhD thesis, Aarhus University. 2019.

[27] Meyer K (2008). Parameter expansion for estimation of reduced rank covariance matrices. Genet Sel Evol..

[28] Madsen P, Jensen J. DMU: a user’s guide. A package for analysing multivariate mixed models. 2013; Version 6, release 5.2. http://dmu.agrsci.dk/. Accessed 12 Sep 2018.

[29] Aguilar I, Misztal I, Legarra A, Tsuruta S (2011). Efficient computation of the genomic relationship matrix and other matrices used in single-step evaluation. J Anim Breed Genet.

[30] VanRaden PM (2008). Efficient methods to compute genomic predictions. J Dairy Sci.

[31] Fischer TM, Gilmour AR, van der Werf JHJ (2004). Computing approximate standard errors for genetic parameters derived from random regression models fitted by average information REML. Genet Sel Evol..

[32] Hiemstra SJ, ten Napel J. Study of the impact of genetic selection on the welfare of chickens bred and kept for meat production. Final report of a project commissioned by the European Commission (DG SANCO 2011/12254); 2013.

[33] Shamim A, Ul Hassan M, Yousaf A, Iqbal MF, Zafar MA, Siddique RM (2015). Occurrence and identification of Emeria species in broiler rearing under traditional system. J Anim Sci Technol..

[34] De Boeck C, Kalmar I, Dumont A, Vanrompay D (2015). Longitudinal monitoring for respiratory pathogens in broiler chickens reveals co-infection of *Chlamydia psittaci* and *Ornithobacterium rhinotracheale*. J Med Microbiol.

[35] Efsa EFSA (2017). The European Union summary report on trends and sources of zoonoses, zoonotic agents and food-borne outbreaks in 2016. EFSA J.

[36] Robertson A (1959). The sampling variance of the genetic correlation coefficient. Biometrics.

[37] Jasouri M, Zamani P, Alijani S (2017). Dominance genetic and maternal effects for genetic evaluation of egg production traits in dual-purpose chickens. Br Poult Sci.

[38] Dana N, vander Waaij EH, van Arendonk JAM (2011). Genetic and phenotypic parameter estimates for body weights and egg production in Horro chicken of Ethiopia. Trop Anim Health Prod.

[39] Maniatis G, Demiris N, Kranis A, Banos G, Kominakis A (2013). Model comparison and estimation of genetic parameters for body weight in commercial broilers. Can J Anim Sci..

[40] Barbieri A, Ono RK, Cursino LL, Farah MM, Pires MP, Bertipaglia TS (2015). Genetic parameters for body weight in meat quail. Poult Sci.

[41] Begli HE, Torshizi RV, Masoudi AA, Ehsani A, Jensen J (2016). Longitudinal analysis of body weight, feed intake and residual feed intake in F_2_ chickens. Livest Sci..

[42] Mebratie W, Shirali M, Madsen P, Sapp R, Hawken R, Jensen J (2017). The effect of selection and sex on genetic parameters of body weight at different ages in a commercial broiler chicken population. Livest Sci..

[43] Mulder H, Bijma P (2005). Effects of genotype × environment interaction on genetic gain in breeding programs. J Anim Sci.

[44] Aguilar I, Misztal I, Johnson DL, Legarra A, Tsuruta S, Lawlor TJ (2010). Hot topic: a unified approach to utilize phenotypic, full pedigree, and genomic information for genetic evaluation of Holstein final score. J Dairy Sci.

[45] Legarra A, Aguilar I, Misztal I (2009). A relationship matrix including full pedigree and genomic information. J Dairy Sci.

[46] Misztal I, Legarra A, Aguilar I (2009). Computing procedures for genetic evaluation including phenotypic, full pedigree, and genomic information. J Dairy Sci.

[47] Hawken R, Sapp R, Okimoto R, Chen J, Borg R, Huang CH (2015). The opportunities and challenges of integrating genomics in a broiler breeding program. Proc Assoc Advmt Anim Breed Genet..

[48] Daetwyler HD, Calus MP, Pong-Wong R, de Los Campos G, Hickey JM (2013). Genomic prediction in animals and plants: simulation of data, validation, reporting, and benchmarking. Genetics.

[49] Daetwyler HD, Villanueva B, Bijma P, Woolliams JA (2007). Inbreeding in genome-wide selection. J Anim Breed Genet.

[50] Gibson JP, Dekkers JCM. Design and economics of animal breeding strategies. Lecture notes—Iowa State University; 2003.

[51] Bijma P (2012). Accuracies of estimated breeding values from ordinary genetic evaluations do not reflect the correlation between true and estimated breeding values in selected populations. J Anim Breed Genet.

[52] van der Heide EM, Lourenco DA, Chen CY, Herring OWL, Sapp RL, Moser DW (2016). Sexual dimorphism in livestock species selected for economically important traits. J Anim Sci..

[53] Mignon-Grasteau S, Piles M, Varona L, De Rochambeau H, Poivey J, Blasco A (2000). Genetic analysis of growth curve parameters for male and female chickens resulting from selection on shape of growth curve. J Anim Sci.

[54] Chapuis H, Tixier-Boichard M, Delabrosse Y, Ducrocq V (1996). Multivariate restricted maximum likelihood estimation of genetic parameters for production traits in three selected turkey strains. Genet Sel Evol..

[55] Mignon-Grasteau S, Beaumont C, Poivey JP, De Rochambeau H (1998). Estimation of the genetic parameters of sexual dimorphism of body weight in’label’chickens and Muscovy ducks. Genet Sel Evol..

[56] Retallick K, Bormann J, Weaber R, MacNeil M, Bradford H, Freetly HC (2015). Genetic variance and covariance components for feed intake, average daily gain, and postweaning gain in growing beef cattle. Kansas Agric Exp Stn Res Rep.

[57] Cardoso FF, Rosa GJ, Tempelman RJ (2007). Accounting for outliers and heteroskedasticity in multibreed genetic evaluations of postweaning gain of Nelore-Hereford cattle. J Anim Sci.

[58] Thompson R (2008). Estimation of quantitative genetic parameters. Proc Biol Sci..

