# Correction to: Use of genomic information to exploit genotype-by-environment interactions for body weight of broiler chicken in bio-secure and production environments

**DOI:** 10.1186/s12711-019-0513-3

**Published:** 2019-11-21

**Authors:** Thinh T. Chu, John W. M. Bastiaansen, Peer Berg, Hélène Romé, Danye Marois, John Henshall, Just Jensen

**Affiliations:** 1 Center for Quantitative Genetics and Genomics, Department of Molecular Biology and Genetics, Aarhus University, 8830 Tjele, Denmark; 2 Wageningen University & Research, Animal Breeding and Genomics, 6709 PG Wageningen, The Netherlands; 3 Faculty of Animal Science, Vietnam National University of Agriculture, Gia Lam, Hanoi, Vietnam; 4 Department of Animal and Aquacultural Sciences, Norwegian University of Life Sciences, 1432 Ås, Norway; 5 Cobb-Vantress Inc, Siloam Springs, AR 72761-1030 USA

## Correction to: Genet Sel Evol (2019) 51:50 10.1186/s12711-019-0493-3

After publication of this work [1], we noticed that there was an error: the formula to calculate the standard error of the estimated correlation ($\widehat{{\rho_{f,r} }}$) between $\hat{u}_{{f_{i} }}$ and $\hat{u}_{{r_{i} }}$ in Appendix on page 12 was mistyped. The correct formula for calculation of the standard error should be:

$$ SE\left( {\widehat{{\rho_{f,r} }}} \right) = \sqrt {\frac{{1 - \widehat{{\rho_{f,r} }}^{2} }}{n - 2}} . $$
